# Probiotics in Allergy and Immunological Diseases: A Comprehensive Review

**DOI:** 10.7759/cureus.55817

**Published:** 2024-03-08

**Authors:** Swapna Vijayan, Venkataramana Kandi, Pratyusha S Palacholla, Reshma Rajendran, Chandrasagar Jarugu, Jayashankar CA, Mundla Pravallika, Shruthi C Reddy, Atul S Sucharitha

**Affiliations:** 1 Pediatrics, Sir Chandrasekhara Venkata (CV) Raman General Hospital, Bangalore, IND; 2 Clinical Microbiology, Prathima Institute of Medical Sciences, Karimnagar, IND; 3 Internal Medicine, Vydehi Institute of Medical Sciences and Research Centre, Bangalore, IND; 4 Public Health, Columbia University, New York City, USA; 5 General Practice, Vydehi Institute of Medical Sciences and Research Centre, Bangalore, IND

**Keywords:** immune system, autoimmune diseases, personalized medicine, prebiotics, probiotics, immunological disorders, allergy

## Abstract

Allergy and immunological disorders like autoimmune diseases are vastly prevalent worldwide. These conditions account for a substantial amount of personal and social burden. Such illnesses have lengthy, uncertain, and spotted courses with unpredictable exacerbations. A definite tendency for improving the overall quality of life of individuals suffering from such diseases is crucial to tackling these diseases, especially through diet or lifestyle modification. Further, interventions like microbiome-based therapeutics such as prebiotics or probiotics were explored. Changes in the microbial population were evident during the flare-up of autoimmune and allergic conditions. The realization that the human microbiome is a central player in immunological diseases is a hallmark of its potential usefulness in therapy for such illnesses. This review focuses on the intricate symphony in the orchestra of the human microbiome and the immune system. New therapeutic strategies involving probiotics appear to be the future of personalized medicine. Through this review, we explore the narrative of probiotics and reaffirm their use as therapeutic and preventive agents in immunological disorders.

## Introduction and background

The World Health Organization (WHO) defines probiotics as "live microorganisms which confer a health benefit on the host when administered in adequate amounts" [1]. The universe of probiotics traverses a broad spectrum of microorganisms beyond the physical or visual scope of our meager comprehension. This has sparked the interest of researchers across the globe. Their integral role in maintaining and nourishing human health has thus been firmly established over the past decades. This is evident from an increasing amount of literature and studies being available in the recent past. The benefit of microbes in preventive primordial management is well-known in healthcare setup and has indeed been on the rise as an unremarkable/surprising gut health promoter [2].

Dysbiosis, a disturbance in the microbial community, is now an undeniable part of the pathogenesis of multiple infectious conditions, autoimmune diseases, and neonatal emergencies [2]. Exploring the potential influence that microbes play on our immune systems has thrown light on the pathophysiology of autoimmune and allergic disorders, paving the way for therapies to be developed and warranting their recognition as functional therapeutic agents.

This multifaceted approach to managing immunological disorders has added another layer to our understanding of the complex web of these diseases and thus improved chances of healing, lower incidences of exacerbations, and increasing periods of remission. An overall improvement in the quality of life for affected patients can be attributed to the efficient usage of microbes in the form of probiotics.

The concept of probiotics in human health and disease could have emerged from the normal human microbial flora, also called microbiome. Humans comprise a balanced ecosystem comprising microorganisms. This ecosystem works closely with our immune system and maintains a nearly impenetrable barrier against potential invaders. These microbial species form an integral part of humans after birth. The human microbiome regulates and maintains multiple internal homeostatic pathways. It guards the fortress of the human body, vigilantly guarding and scouring for intruders, including pathogens, once identified, to be confined and eliminated. The dysregulation in the human microbiome results in microbial translocation and gene expression at the post-transcriptional level, producing microbial metabolites that interact with cellular receptors such as toll-like receptors (TLRs) and G‐protein-coupled receptors (GPCRs) [3]. This avalanche sequence further contributes to the emergence of autoimmune disorders in predisposed individuals.

Immunonutrition is an umbrella term encompassing the maintenance of internal homeostasis by nutrition. This shields against adverse effects of immunity, infection, inflammation, and injury. The main immunonutrients include amino acids, essential fatty acids, probiotics, and antioxidants [4].

Gut dysbiosis causes an imbalance between pro- and anti-inflammatory systems and disturbing normal homeostasis. Thus, a comprehensive understanding of the relationship between dysbiosis and autoimmune diseases is critical for providing novel insights into developing microbiota-based therapeutic approaches for combating refractory diseases like autoimmune and allergic conditions [5].

**Figure 1:**
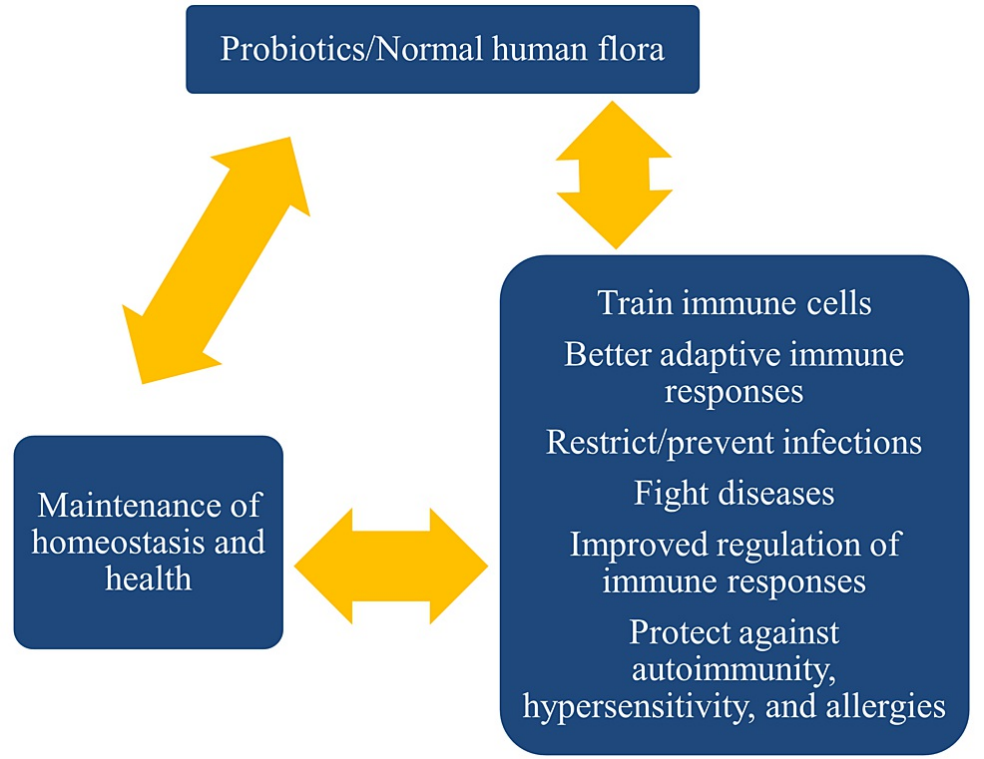
The roles played by probiotics and normal human microbial flora Image Credit: Venkataramana Kandi

The probiotics and human microbiome work by restricting the colonization of invading microbes. Besides, the presence of these microbes facilitates recognition by the immune system and contributes to the clearance through neutralization by specific antibodies. The host-microbial flora trains immune cells to function during adaptive immune responses, prevent microbial colonization and infection, regulate immune responses, fight diseases, and protect against autoimmunity, hypersensitivity, and allergic reactions [6] (Figure 1).

## Review

Several immunological disorders affect people, including food allergies, allergic rhinitis (AR), atopic dermatitis (AD), asthma, Sjogren's syndrome (SS), Crohn's disease (CD), coeliac disease, type 1 diabetes mellitus (T1DM), myasthenia gravis (MG), and rheumatoid arthritis (RA), among others. These immunological disorders affect different cells and organs of the body, like lungs, skin, eyes, mouth, joints, pancreatic islet cells, nerves, and muscles. Interestingly, all these disorders stem from disturbances in the immune responses. Some are due to hypersensitivity, wherein the immune system demonstrates an exaggerated response to antigens or allergens like food and dust. Few other disorders arise from autoimmune conditions wherein the immune system reacts with the host (cells or tissues) by producing autoantibodies. This is evident in T1DM patients who develop autoantibodies against pancreatic islet cells, which get destroyed, leading to the disease. 

**Figure 2:**
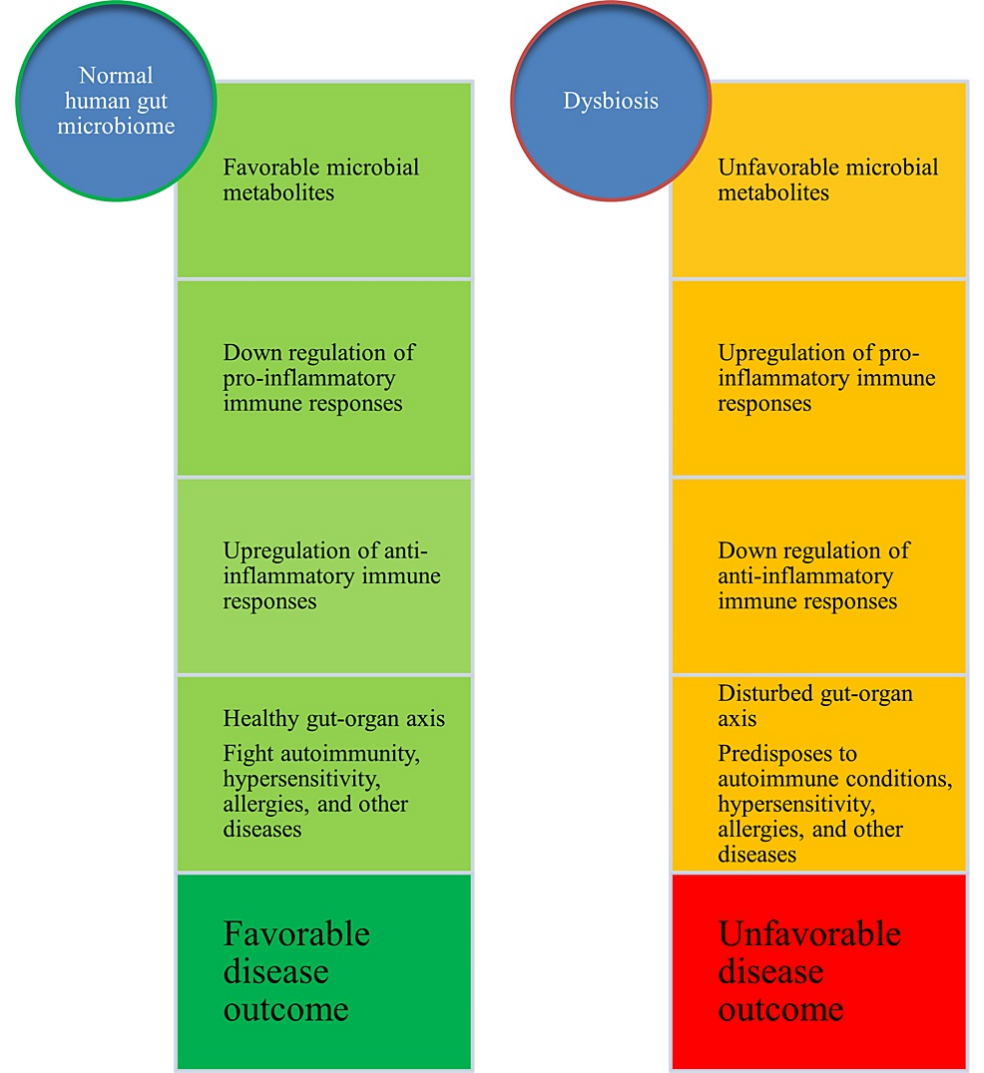
Effects of human microbiome and dysbiosis on health, immune responses, and disease Image Credit: Venkataramana Kandi

Recently, there has been an increased interest in the potential role of the gut microbiome in influencing the health of different organs/systems of the human body. This is further supported by theories like the gut-brain axis, gut-lung axis, gut-liver axis, gut-kidney axis, gut-skin axis, gut-brain-skin axis, and gut-organ axis [7-9]. All these relationships have been proposed, linking the role of the gut microbiome and its metabolic byproducts. These include hormone-like compounds, single-chain fatty acids (SCFAs), and lipopolysaccharides (LPS). Most bacterial metabolites directly influence the immune system and its responses during infections, hypersensitivity, and autoimmune disorders. Favorable gut microbiome results in the downregulation of pro-inflammatory responses, and dysbiosis/disturbances in the gut microbiome cause upregulation of inflammatory immune responses. This potentially damages the host cells, tissues, and organs. Therefore, a healthy gut microbiome could be a plausible solution to restricting and minimizing host damage during infection. A favorable gut microbiome composition could avoid autoimmune-based immunological responses and protect people against autoimmunity and its adverse effects. Besides, a healthy gut microbiome could result in favorable disease outcomes compared to a dysbiosis status wherein people are predisposed to unfavorable disease outcomes (Figure 2).

A promising study by Abdulkadir et al. in 2016 demonstrated probiotic species successfully colonizing the preterm neonatal gut, reducing the relative abundance of potentially pathogenic bacteria and influencing gut functioning. *Bifidobacterium *(but not *Lactobacillus*) colonized the gut in the long term, throwing favorable light on therapeutically administered probiotics and their effects on gut microbial communities in early infancy [10]. The human microbiome was influenced by delivery through cesarean section, perinatal antibiotics, and formula feeding. Consequently, these practices become risk factors for increased chances of developing metabolic and autoimmune diseases after birth [11]. We comprehensively discuss the role of probiotics in treating and managing some common allergies, hypersensitivity reactions, and autoimmune disorders. 

Food allergy

Food allergy is an immunological reaction that follows exposure to certain foods and leads to adverse health effects. One of the most direct ways to improve gut microbiota is through the intake of probiotics, which play a vital role in treating food allergies. Most studies have been conducted in infants and children, considering the gut microbiome alters over the years. It is more advantageous for them as they not only alleviate food allergies but can also treat food intolerance. This is supported by the results of a recent study in individuals with non-coeliac sensitivity where enrichment with a multi-strain probiotic that consisted of *Lactobacillus*, *Bacillus coagulans*, and *Saccharomyces boulardii *was able to alleviate the symptoms [12].

The preeminent approach in handling such allergies is avoidance of the causative foods. Consequently, it is crucial to explore alternative modes of treatment, such as probiotics. In addition to the ability of probiotics to relieve food allergies by adhering to intestinal epithelial cells and modulating gut flora, their protective factor to human health confirms their role as capable restorative agents. The most frequently used probiotics to treat food allergies include *Lactobacillus *and *Bifidobacterium.*

The *Lactobacillus *strains frequently used include *Lactobacillus rhamnosus *and *Lactobacillus murinus*. The former was found effective in treating cow milk-related allergies in infants. Therefore, this bacterium is being added to infant formulas. *L*.* murinus *can upregulate the performance of both thymus (T) helper cells 1 (Th1) and T regulatory (Treg) cells, which impact the production of immunoglobulin (Ig) E (IgE) antibodies. This was achieved by preventing class switching from IgM to IgE during allergies [10-12].

Other beneficial bacteria like *Bifidobacterium longum *and *Bifidobacterium lactis *have also been discovered to help prevent food allergies. *Bifidobacterium longum *augments the effects of IgE_TRAP_ through the obliteration of mast cells by apoptosis. IgE_TRAP_ comprises a FcεRIα (tetrameric receptor complex that binds to fragment of crystallization (Fc) portion of the epsilon (ε) heavy chain of IgE antibody) extracellular domain and an IgD/IgG4 hybrid Fc domain forming a fusion protein [13]. On the other hand, *Bifidobacterium lactis *was noted to exert its effects on T cells. *B*. *lactis *treatment substantially elevated the forkhead transcription factor family-related protein (FoxP3) expression and transforming growth factor-beta (TGF-β) related to Treg cells. Simultaneously, this reduced the activities of interleukin (IL)-17A and IL-23 related to Th17 immune responses [14].

AR

AR has been increasingly affecting individuals over the years. It results in the inflammation of the nasal cavity due to the activation of mast cells and the subsequent release of histamine and other vasoactive molecules. AR is generally triggered following exposure to pollen, dust, etc. Due to its complications and unsatisfactory treatment, many treatment modalities have been explored.

Recent studies have determined the beneficial role of probiotics in treating and managing AR. Along with improving the quality of life, they also shorten signs and symptoms. Commonly used probiotics include *Lactobacillus *and *Bifidobacterium*. The role of probiotics in treating AR is defined by their ability to activate dendritic cells, the most efficient antigen-presenting cells (APCs). Furthermore, they can exercise their effects on cellular and molecular pathways by regulating the production of cytokines. Probiotics can significantly impact immunoglobulin regulation by improving IgG antibodies and decreasing the levels of IgE antibodies. They also enhance the production of inflammatory cytokines like interferon-gamma (IFN-γ), IL-6, and tumor necrosis factor-alpha (TNF-α) to prevent infections [13,14]. Recently, a mixture of *Lactobacillus rhamnosus *GG (LGG), named after scientists Sherwood Gorbach and Barry Goldwin, who first isolated the strain in humans, IL-2, and green fluorescent protein (GFP) as a fusion protein (LGG-IL-2-GFP) were employed to view the bacterial uptake and the immune response activated by oral immunization. The results from this experiment showed that in addition to expressing an antigen, LGG could further produce an effective immune response to the antigen that includes enhanced IL-2 secretion [15].

It was found that the ramifications are specific to each probiotic type. Among the drawbacks, the risk of probiotics transferring genes to the host was supported by the evidence of *L*. *reuteri *and *L*. *plantarum *carrying antibiotic-resistance genes [15]. It was also observed that supplementation with LGG caused septicemia in children with short-bowel syndrome [16]. Similar complications were witnessed in immunodeficient populations as probiotics contributed to sepsis through bacteremia and fungemia. This causes probiotic usage in AR to be challenging and scarcely recommended.

AD

AD is a persistent hypersensitivity disorder of the skin triggered by allergens that are harmless to nonatopic individuals [17]. It commonly presents as pruritus, dry skin, and rashes. AD predominantly affects infants and young children, with 75% of cases appearing before the age of five years. The higher occurrence in urban areas can be attributed to reduced exposure to microbes due to urbanization. Factors such as limited contact with farm animals and pets, increased use of antibiotics and vaccines, and improved infant hygiene contribute to disturbances in the child's gut microbiome and immune system development. Maternal diet during pregnancy and after birth also influences the child's gut microbiome. An imbalance in the microbiome leads to a persistent abnormal immune response dominated by Th2 cells in newborns, resulting in the excessive production of pro-inflammatory cytokines against common environmental allergens. AD is associated with mutations in the filaggrin gene (FLG). FLG encodes a protein crucial for maintaining skin barrier integrity by retaining moisture and protecting against environmental allergens [18].

Administering probiotics is one of the most effective ways to restore the gut microbiome. The SCORAD (Severity Scoring of Atopic Dermatitis) index is a widely used parameter to gauge the effectiveness of treatment. A study conducted by Isolauri et al. on infants who manifested AD were breastfed and weaned onto probiotic-supplemented formulas containing *Bifidobacterium lactis* (Bb-12) and *Lactobacillus *strain GG (American Type Culture Collection (ATCC)-53103). There was a significant improvement in the skin condition and reduced SCORAD indices in infants who weaned through probiotics [19].

Due to insufficient evidence, the World Allergy Organization (WAO) does not recommend the use of probiotics in pregnant and lactating women to prevent AD [20]. In a study by Huang et al., who included children aged between one and 18 years, an improvement in the SCORAD index was noticed upon supplementation with *Lactobacillus fermentum*, *Lactobacillus salivarius*, and a mixture of different strains [21]. Probiotics were also known to improve symptoms in adults with AD. Intake of a combination containing *Lactobacillus salivarius *(LS01) and *Bifidobacterium* *breve *(BR03) improved the SCORAD index [22].

However, the exact mechanism of action of these probiotics remains unknown. Probiotics suppress the Th2-mediated immune response and enhance the Th1-type immune responses. This inhibition of cytokine release reduces inflammation. Despite this knowledge gap, the tangible results witnessed in some clinical trials should encourage us to explore the potential of probiotics as adjuvant therapy in treating and managing AD.

While steroids have long been widely used in the treatment of AD, their advantages come with a set of drawbacks that cannot be overlooked, prompting the search for alternative solutions. Steroids may provide temporary relief, but their limitations become apparent over time. They lead to skin thinning, discoloration, and increased susceptibility to infections as they suppress the immune system. In the face of these limitations, a quest for safer and more sustainable solutions ensues. Probiotics emerged as a promising alternative, offering a softer approach that nurtures the body's natural defenses and restores the balance of the human microbiome.

Asthma

Asthma is a chronic disease of the lungs with widespread occurrence among children and adults. It results in the inflammation of the pulmonary airways and bronchial hyperresponsiveness due to multiple factors. Asthma causes lower airway obstruction and is a reversible condition [23]. Wheezy respiration, coughing, chest tightness, and shortness of breath are typical manifestations of asthma.

Dysbiosis is evident in asthma, which causes altered immune responses within the guts and impacts distant organs such as the lungs. The concept of the gut-lung axis originated from the observation that alterations in the intestinal environment can impact the development and progression of various lung diseases and vice versa. Although the precise mechanism is not yet fully understood, a commonly accepted hypothesis suggests that mediators originating from intestinal epithelial cells like immune cells, microbial structural components, and microbial metabolites travel through the bloodstream and induce alterations in immune responses within the lungs [24].

Probiotics are gaining increasing recognition as a therapeutic approach for allergic conditions like asthma. They enhance the Th1 immune response, thereby downregulating IgE production, alleviating airway inflammation, and bolstering the immune defense against respiratory infections. Probiotics interact with intestinal epithelial cells and immunocompetent cells through TLRs, triggering the production of immune mediators, cytokines, and chemokines. Additionally, they activate Treg cells, releasing IL-10, a pivotal anti-inflammatory cytokine involved in immune regulation. Probiotics also modulate the intestinal microbiota, inhibiting the growth of potentially harmful bacteria within the gut [25].

Supplementation with the *Lactobacillus gasseri* strain showed a significant reduction in TNF-α, IFN-γ, IL-12, and IL-13. Pulmonary function and peak expiratory flow rate (PEFR) increased significantly [26]. Studies conducted by PROGRAM (Probiotics in Paediatric Asthma Management) have shown clinical evidence suggesting that *Bifidobacterium breve *B632 (DSM-24706) and *Ligilactobacillus salivarius *LS01 (DSM-22775) may prevent asthma exacerbations in children [27]. Miraglia Del Giudice et al., in their study, observed that a *Bifidobacterium *mixture containing the strains *B*. *longum *BB536, *B*. *infantis *M-63, and *B*. *breve *M-16V significantly improved symptoms of children with intermittent asthma [28]. A multi-modal treatment approach with probiotics can be promising to manage chronic disorders such as asthma.

SS

SS is an autoimmune disorder characterized by dry eyes and mucous membranes. It results from autoimmune-mediated lymphocytic infiltration of the salivary, lacrimal, and exocrine glands. The gut microbiome plays a significant, if not central, role in the pathogenesis of SS. The ocular-gut axis comprises the relationship between human gut microbiota and the immune regulation of the eyes. Intestinal dysbiosis has a connection with the severity of ocular mucosal diseases in SS. This can be confirmed by the research carried out by Moon et al., who reported that the relative abundance of microbial species like *Bacteroides*, *Actinobacteria*, and *Bifidobacterium *in the gut of people suffering from SS was significantly associated with dry eye symptoms [29]. Greater relative abundances of *Pseudobutyrivibrio*, *Escherichia*, *Shigella*, and *Streptococcus *while reduced relative abundances of *Bacteroides*, *Parabacteroides*, *Faecalibacterium*, and *Prevotella *were noted with SS, compared to controls. This dysbiosis contributes to the imbalances in Th1- and Th17-type immune responses, polarization of Tregs, and production of SCFAs [30].

The association between gut bacteria and autoimmune disease is likely a two-way communication. The gut microbiome abnormalities can lead to systemic inflammation. Conversely, systemic inflammation can preferentially deplete beneficial gut bacteria and promote the growth of bacteria with potential pathogenic properties. This dysbiosis is associated with clinical and laboratory markers of disease activity in SS and could potentially be used as a predictor in the future [31].

CD

CD is an autoimmune intestinal disorder caused by a dysfunctional interaction between the microbiota and the immune system [32]. It is a type of irritable bowel disease (IBD), which is linked closely with interactions between genetic predisposition, environmental factors, and mucosal immunity [33]. CD is more common among older adults and is characterized by a transmural granulomatous inflammation, occurring discontinuously and giving rise to skip lesions in the intestines. Although it affects the entire intestine, the caecum is primarily involved, while perianal fistulas are common [34].

Symptoms generally include diarrhea, abdominal pain, blood or mucus in feces, perineal pain, weight loss, secretion, and irritation due to perianal fistulas. Extra-intestinal manifestations of the disease, such as arthritis, uveitis, and rash, are also observed. Thus, the changes in the intestinal microbiome due to CD interfere with the symbiotic relationship between the immune system and the intestinal microbiome, leading to pathological consequences [35].

Corticosteroids, aminosalicylates, and other immunosuppressive agents are the mainstay of treatment for the induction and maintenance of remission. Probiotics may be used to manage the microbiome. Hence, they were considered a potential adjuvant therapy for CD. In particular, the role of yeast (fungus) *Saccharomyces boulardii *helped maintain remission and bowel sealing [36].

*Faecalibacterium prausnitzii *appears more helpful in treating and managing CD, suggesting that counterbalancing dysbiosis using *F*. *prausnitzii *as a probiotic is a promising strategy [37]. There is evidence that probiotics benefit other gastrointestinal conditions, such as IBD and ulcerative colitis. The progression of CD causes increased mucosal permeability, which perpetuates intestinal inflammation. It was previously observed that the administration of probiotics in the early stages of CD could potentially ameliorate symptoms by stabilizing the intestinal barrier [38].

Coeliac disease

Coeliac disease is a chronic autoimmune enteropathy arising out of gluten intolerance. It has been shown to have an intrinsic association with altered gut microbiota [39]. Elevation in the proportions of various bacterial species, including *Firmicutes *and *Bacteroides, *and reduction in the populations of gluten-proteolytic bacteria, such as *Bifidobacterium*, *Lactobacillus*, and *Rothia *species, have been reported in patients with the active coeliac disease [40,41]. This, in turn, augments the balance between bacterial LPS and metabolites such as SCFAs. LPS increase causes activation of various intraepithelial lymphocytes (IELs), which trigger a cascade of antimicrobial peptides (AMPs) and mucin production. Treg and dendritic cells are also activated and produce IL-10 and retinoic acid, which induces an inflammatory response in the lamina propria [41,42].

In a recent study by Medina et al., certain species of *Bifidobacterium *were found to have a protective role in coeliac disease. This was confirmed by the presence of higher numbers of *B*. *longum *in people without coeliac disease compared to those found in active and non-active coeliac disease patients [43]. Another study showed that prolyl endopeptidases from *Sphingomonas capsulata*, *Flavobacterium meningosepticum*, *Myxococcus xanthus*, and *Aspergillus niger *could be pursued as drug candidates for the enzymatic treatment of gluten in coeliac disease patients. Prolyl endopeptidases target the conformationally constrained peptide bonds at the C-terminal [44]. An agreement was reached among physicians and researchers about the role of probiotics in alleviating gastrointestinal symptoms, especially in symptomatic coeliac disease patients. This strategy is being tested by clinical trials instead of being enlisted as a remission strategy [45].

T1DM

T1DM is a rarer form of diabetes that results from an autoimmune-mediated pancreatic beta-cell destruction, which causes insulin deficiency. The occurrence of T1DM may be synonymous with changes in the gut microbiome and immune regulation. Gut barrier dysfunction, altered gut ultra-structure and permeability, inflammation of the duodenal mucosa, and dysbiosis are associated with T1DM. When synergized with environmental vulnerabilities early in life, these factors may influence abnormal gut colonization and disease progression [46].

A relationship between good neonatal probiotic supplementation leading to reduced pancreatic islet cell autoimmunity and decreased risk of devolving T1DM was previously elucidated [47]. Another intriguing association was found between cesarean birth and increased risk of developing T1DM. This was established following evidence of altered gut microbiota and immune responses among neonates born through cesarean sections [48]. It was observed that delivery by cesarean section is associated with a more than twofold increase in the risk of developing T1DM. Notably, this increase was not due to higher numbers of children developing pancreatic islet cell autoimmunity but instead by a faster progression from the onset of autoimmunity to overt disease [49].

Importantly, lactic acid bacteria belonging to the genera *Bifidobacterium *and *Lactobacillus *were transferred from mother to child during natural birth and are essential for catabolizing milk oligosaccharides [49]. These bacterial species are also known to support intestinal barrier integrity, produce anti-inflammatory SCFAs, and are often used as probiotic supplements [50]. Modulating the gut microbiota normalizes the endogenous gut, maintains pancreatic health, and lowers plasma TLR-4 ligand levels. This confirms that probiotic supplementation may lower systemic inflammation in unaffected siblings of T1DM patients [51]. In a landmark study, The Environmental Determinants of Diabetes in the Young (TEDDY), that included six clinical centers (three in the United States (Colorado, Georgia, and Washington) and three in Europe (Finland, Germany, and Sweden)), it was found that exposure to probiotic supplements during neonatal period (first 28 days of life) could potentially reduce the risk of T1DM in predisposed children [52].

MG

MG is an autoimmune disorder that affects the neuromuscular junction. It is characterized by impairment in the acetylcholine receptor (AChR). The autoantibodies bind to AChR, blocking the postsynaptic region. Individuals with MG showed a distinctive composition in the oral and gut microbiota, with a quintessential increment in *Streptococcus *and *Bacteroides, *a decline in *Clostridia*, and a reduction in SCFAs compared with age-matched controls. Since MG is chorionic and remains lifelong, several treatment modalities have been tried since side effects have been seen with chronic usage of many first-line medications.

Probiotic usage in MG can lower autoantibody production and lessen inflammatory cytokine expression in conjunction with decreasing anti-AChR antibody serum titers. A previous study demonstrated that the five-times-per-week administration of IRT5, a probiotic mixture comprising *Streptococcus thermophilus*, *Lactobacillus reuteri*, *Bifidobacterium bifidum*, *Lactobacillus acidophilus, *and *Lactobacillus casei*, starting two weeks before Experimental Autoimmune Myasthenia Gravis (EAMG) induction, improved EAMG progression through the inhibition of AChR-reactive lymphocyte proliferation, lowered inflammatory cytokine expression, and anti-AChR antibody production [53]. Specific probiotic strains can escalate the generation of anti-inflammatory cytokines and Treg cells, which help to control inflammation [54].

Gut microbiota dysbiosis is seen as a driving factor in autoimmune conditions by increasing the permeability of the intestinal mucosal barrier and accentuating inflammatory responses. Through experimentation in autoimmune diseases like T1DM and RA, it was observed that probiotics balance local and systemic inflammatory immune responses and maintain gut microbial homeostasis [55]. Probiotics have an indispensable therapeutic effect on gut regulation and are a leading choice to treat and manage these conditions in the forthcoming years.

RA

RA is a chronic autoimmune disease affecting approximately 1% of the global population [56,57]. The pathogenesis of the disease is due to autoimmune dysfunction and malfunctioning of signaling networks that result in impaired tissue repair processes, leading to organ damage, vascular damage, and joint capsule degeneration, the most frequent clinical manifestation. The onset of RA is usually insidious and presents with fever, malaise, and generalized weakness, which later progresses to generalized inflammation and swelling. RA predominantly affects the musculoskeletal system, and the articular manifestations include polyarthritis affecting the hands and feet, polyarthralgia, and progressive articular degeneration [58]. The extra-articular manifestations of RA include normocytic normochromic anemia, chronic leg ulcers, osteoporosis, rheumatoid vasculitis, and skin manifestations. Although less frequently observed in clinical practice, RA also presents with a lung picture of exudative pericarditis, which can cause pleural effusion [59].

In RA, the dysbiosis of the gut microbiome is at the crux of altered systemic response and inflammation, leading to clinical manifestations [60]. Previous studies have demonstrated a significant decrease in microbial diversity in RA patients compared to the gut microbiota of healthy persons [60,61]. The decreased diversity of the gut microbiome was further linked to increased disease duration [60-62].

Several mechanisms by which gut microbiota are associated with arthritis have been proposed. These include regulating the host's immune system (triggering T-cell differentiation), activating APCs through an effect on TLRs or NOD-like receptors (NLRs), aiding in the enzymatic citrullination of peptides, molecular mimicry of antigens, and increasing the intestinal mucosal permeability [62-67]. These pathways coalesce to cause an imbalance in the Th17/Treg cell ratio, and this local immune response results in systemic autoimmunity. The existing literature suggests that gut microbiome could contribute to or prevent the expansion of autoimmunity and inflammation during the preclinical and clinical phases of RA.

*Faecalibacterium*, a member of the phylum *Firmicutes, *is the most ubiquitous commensal in a healthy gut. *Faecalibacterium *is responsible for the production of butyrate, a key gut metabolite responsible for maintaining intestinal immune homeostasis [61]. The depletion of various butyrate-producing species like *Faecalibacterium *and *Flavobacterium *has been closely linked with the development of RA. Additionally, there was an increase in *Lactobacillales *compared to a healthy human gut [62].

The sequence of events occurring in RA confirms the increase in Gram-positive bacteria and depletion of Gram-negative bacteria [64]. The dysbiosis of the gut microbiome leads to the downregulation of TLRs while simultaneously activating pattern recognition receptors (PRRs), which sets into motion a systemic inflammatory response [57,60]. The subsequent imbalance between anti-inflammatory and pro-inflammatory cytokines, namely, IL-1β, TNF, IFN-γ, IL-6, IL-12, and IL-17, contributes to the pathogenesis of RA [63,67].

Probiotics play a pivotal role in reducing the inflammatory manifestations in RA by significantly reducing inflammatory indicators such as C-reactive protein (CRP) [67]. The gut seems to have an increased permeability towards probiotics, which reduces intestinal inflammation [64]. Currently, probiotic supplementation with *L*. *casei *is at the forefront of probiotics used in therapy for RA [64,67]. *L*. *casei *has shown exceedingly high tolerance to acidic gut conditions while at the same time exhibiting resistance to intestinal bile. It* *is, therefore, the most beneficial probiotic to treat RA [65,67].

A systematic review and meta-analysis investigating the effectiveness of probiotic supplementation in RA underlined that the trials in which a significant reduction of CRP was achieved used *L*. *casei *[62,65,67]. A study conducted by Vaghef-Mehrabani et al. among patients diagnosed with inactive to moderate RA treated with disease-modifying anti-rheumatic drugs (DMARDs) and glucocorticoids for a minimum of three months showed a significant decrease in pain levels when given daily capsules of *L*. *casei *(>10^8^ colony-forming units (CFU)/capsule) for eight weeks. This was measured by a 43.96% decrease in the Visual Analogue Scale (VAS) compared with the placebo group that was given only maltodextrin capsules. Additionally, decreased levels of pro-inflammatory cytokines (TNF, IL-6, and IL-12) and increased activities of anti-inflammatory cytokines like IL-10 were seen in the probiotic group [65,67]. Such findings only point towards a promising future for the role of probiotics as a standard inclusion/adjunct in therapeutic regimens for RA and other autoimmune diseases [67-69].

## Conclusions

Having reviewed the multitude of literature, the emergent use of probiotics as an adjunct therapeutic intervention for induction and maintenance of remission in the spectrum of autoimmune diseases speaks for itself. *Bifidobacterium*,* Lactobacillus*, and* Saccharomyces *species are some examples of probiotics, which when given individually or as a mixture in different combinations facilitate remission of these diseases. The seamless ability of probiotics to enrich self-sustenance and their enormous value have cemented their role as keys to good gut health and immunity. Since the human microbiome acts as a regulator and protector of interior homeostasis, establishing a healthy variety of microflora within human bodies is crucial to lowering immune aberrations. The increasing attention towards probiotic supplements as part of a daily routine, not only by the public for their health-promoting effects but also by clinicians in light of their efficacy as supplements and therapeutic properties, is paving the way for more holistic approaches in the fields of immunology and immunological diseases.
